# The endothelium: gatekeeper to lung ischemia-reperfusion injury

**DOI:** 10.1186/s12931-024-02776-4

**Published:** 2024-04-18

**Authors:** Huy Q. Ta, Maniselvan Kuppusamy, Swapnil K. Sonkusare, Mark E. Roeser, Victor E. Laubach

**Affiliations:** 1 Department of Surgery, University of Virginia, P. O. Box 801359, Charlottesville, VA 22908 USA; 2 Robert M. Berne Cardiovascular Research Center, University of Virginia, Charlottesville, VA 22908 USA; 3 Department of Molecular Physiology and Biological Physics, University of Virginia, Charlottesville, VA 22908 USA

**Keywords:** Endothelial barrier dysfunction, Ischemia-reperfusion injury, Lung transplantation, Glycocalyx, Ion channels, TRPV4, Inflammation, Oxidative stress

## Abstract

The success of lung transplantation is limited by the high rate of primary graft dysfunction due to ischemia-reperfusion injury (IRI). Lung IRI is characterized by a robust inflammatory response, lung dysfunction, endothelial barrier disruption, oxidative stress, vascular permeability, edema, and neutrophil infiltration. These events are dependent on the health of the endothelium, which is a primary target of IRI that results in pulmonary endothelial barrier dysfunction. Over the past 10 years, research has focused more on the endothelium, which is beginning to unravel the multi-factorial pathogenesis and immunologic mechanisms underlying IRI. Many important proteins, receptors, and signaling pathways that are involved in the pathogenesis of endothelial dysfunction after IR are starting to be identified and targeted as prospective therapies for lung IRI. In this review, we highlight the more significant mediators of IRI-induced endothelial dysfunction discovered over the past decade including the extracellular glycocalyx, endothelial ion channels, purinergic receptors, kinases, and integrins. While there are no definitive clinical therapies currently available to prevent lung IRI, we will discuss potential clinical strategies for targeting the endothelium for the treatment or prevention of IRI. The accruing evidence on the essential role the endothelium plays in lung IRI suggests that promising endothelial-directed treatments may be approaching the clinic soon. The application of therapies targeting the pulmonary endothelium may help to halt this rapid and potentially fatal injury.

## Introduction

While lung transplant rates continue to rise annually, the outcomes of lung transplantation are the worst of any solid organ transplant [1]. Approximately 50% of patients die within 5 years of transplant, and ~ 67% within 10 years, despite progress in lung preservation, surgical management and immuno-suppression therapies [2]. The success of lung transplantation is limited by a high rate of primary graft dysfunction (PGD) due to ischemia-reperfusion injury (IRI), which is a rapid and complex sterile inflammatory response characterized by dramatic elevations of extracellular adenosine triphosphate (ATP), oxidative stress, robust innate immune responses, rapid and potent release of proinflammatory signals, vascular permeability, edema, and endothelial and epithelial barrier dysfunction after transplant [3–6].

Lung IRI not only leads to PGD after transplantation, but IRI is also a risk factor for chronic lung allograft dysfunction, the major cause of mortality in recipients [7, 8]. 29% of transplant patients with IRI will die within 90 days, compared to 5% of recipients without [9]. Furthermore, those transplant patients with IRI and PGD will experience protracted mechanical ventilation and inpatient hospital care, as well as increased risk of multi-organ failure [10]. Currently no therapeutic agents are clinically available to prevent IRI, and treatment strategies are limited to maintaining function.

**Fig. 1:**
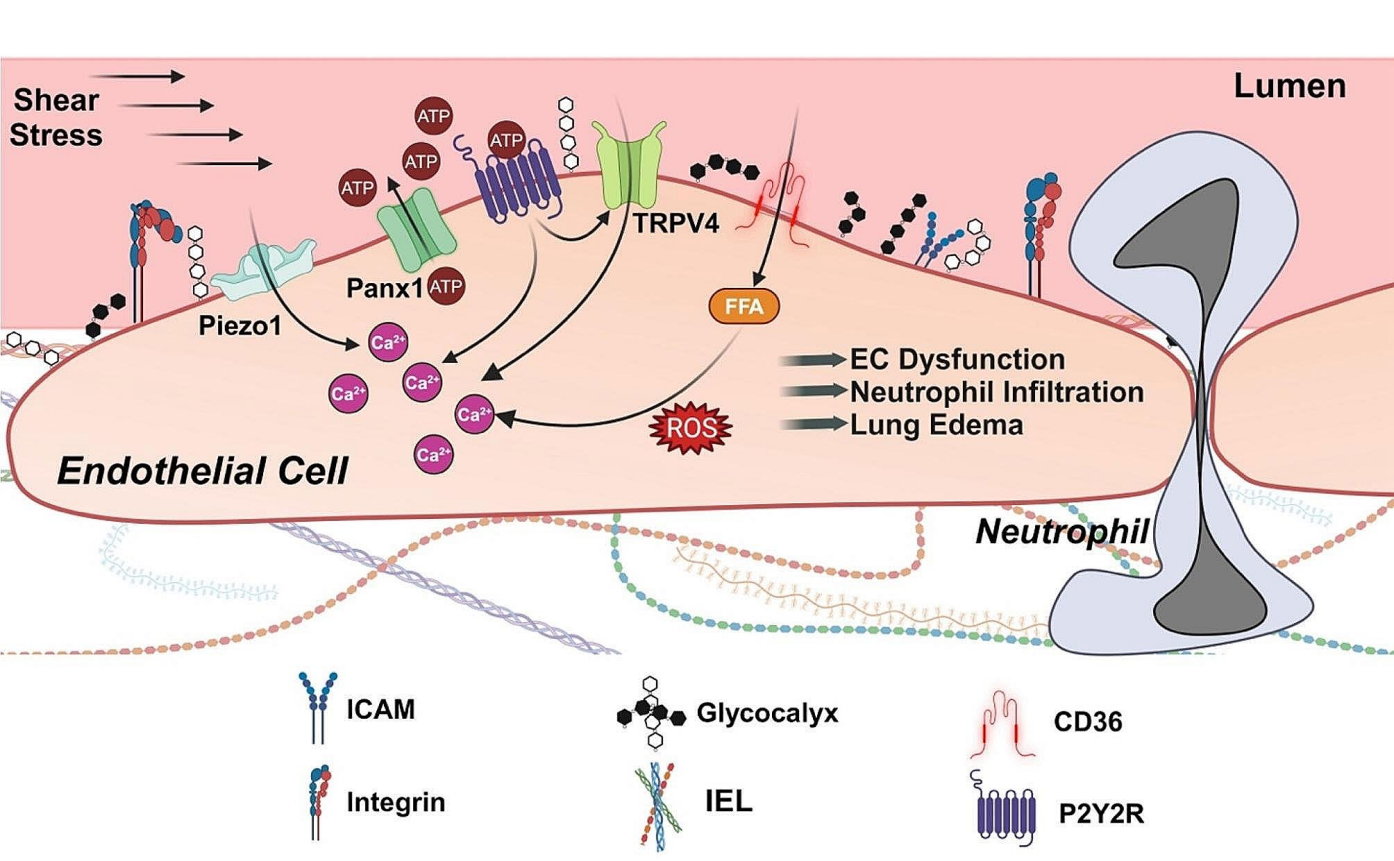
**Endothelial events leading to lung IRI**. Reperfusion can increase fluid shear stress and activate mechanosensitive Piezo1 channels at the cell membrane. Piezo1 channels are a crucial Ca^2+^ entry pathway in endothelial cells. IR also increases extracellular ATP levels in the lung via Panx1 channels. Extracellular ATP can activate purinergic receptors, further increasing cytosolic Ca^2+^ levels. ATP activation of purinergic P2Y2 receptors (P2Y2R) leads to increased activity of TRPV4 channels, another mechanically activated Ca^2+^ entry pathway on endothelial cell membrane. FFA transporter CD36 elevates cytosolic FFA levels, in turn increasing Ca^2+^ influx and ROS production and triggering a cytokine storm. These pro-inflammatory intracellular signaling events dismantle intercellular junctions and promote neutrophil infiltration, increased capillary fluid extravasation, and lung edema formation. Glycocalyx masks the endothelial surface adhesion molecules (ICAM, VCAM, P- and L-selectin) and has a protective effect against IR-mediated deleterious events. Integrins at the cell surface are also involved in increased vascular permeability after IR. IEL, internal elastic lamina; FFA, Free fatty acid; CD36, Cluster of differentiation 36

The pulmonary endothelium is a primary target of IRI, and a key hallmark of IRI is endothelial cell (EC) dysfunction, which leads to increased pulmonary fluid accumulation (edema), impaired gas exchange, and decreased lung compliance [11]. IR stimulates the production of proinflammatory chemokines, cytokines, damage-associated molecular patterns (DAMPs), and reactive oxygen species (ROS) from a variety of cells including ECs, resulting in EC swelling and detachment from the basement membrane. This EC-mediated inflammatory response results in increased vascular permeability, which in turn activates innate immune cells and promotes leukocyte adherence and transmigration. Furthermore, these immune cells release additional inflammatory mediators that further damages the endothelium, ultimately leading to lung injury and graft failure. The leukocyte-endothelial interface is regulated by a complex signaling network of proteins, enzymes, receptors, and channels that perform crucial functions in the endothelium. While the mechanisms of lung IRI are not fully understood, a plethora of studies have determined that EC injury plays a critical role, and many of the regulators that influence endothelial dysfunction and IRI have been identified, providing promising therapeutic strategies to prevent IRI (Fig. 1). Therefore, in this review, we discuss the impact that the endothelium has on lung IRI, with a focus on the more recent and targetable mediators of EC dysfunction. We also present potential clinical strategies for targeting the endothelium for the treatment of IRI.

## Endothelium

The vascular endothelium was once believed to be an inactive, stagnant structure. However, it is now appreciated as a highly specialized metabolically active organ that modulates several fundamental physiological, immunological, and synthesizing processes, such as leukocyte extravasation, intravascular clotting, vasomotor tone, inflammation, barrier permeability, production of chemokines, cytokines, growth factors, and ROS, as well as expression of receptors, signaling molecules, and adhesion molecules [12–15]. Lining the innermost layer of blood vessels, the vascular endothelium consists of a monolayer of mesenchyme-derived ECs, subcellular extracellular matrix (ECM) proteins, and lumenal glycocalyx. The glycocalyx structure functions as a permeable barrier segregating blood from lung tissue, regulating trafficking of proteins, nutrients, leukocytes, and fluids [16, 17]. Furthermore, components within the EC membrane can also modulate intracellular signaling pathways that regulate fundamental biological functions, such as metabolism, gene expression, and cell structure, by sensing changes in fluid shear stress and hemodynamic pressure [18, 19].

The integrity of the endothelium is regulated by intercellular junctions (tight junctions and adherens junctions) between neighboring ECs [16]. These junctions maintain barrier function and modulate signal transduction through interactions with cytoskeletal microtubules and actin microfilaments in response to forces exerted on the endothelium [20]. Cell surface integrins connect the monolayer of ECs at focal adhesion plaques. Although the integrity of the endothelium depends on both tight and adherens junctions, vascular permeability and subsequent edema develops mainly as a result of dysfunction of tight junctions [12]. Since interendothelial junctions are also covered by the endothelial glycocalyx, dysfunction of the glycocalyx can also cause leakage [21].

Given its roles in mechanosensation, secretion, and metabolism, it is not surprising that the endothelium is highly vulnerable to the damaging effects of IR [3]. Ischemia initiates a proinflammatory response within the endothelium that primes and sensitizes it to additional injury upon reperfusion, where a robust local and systemic inflammatory response extensively damages the integrity of the endothelial barrier through the formation of gaps and expression of transmembrane ion channels. Oxidative stress, ROS, and reactive nitrogen species (RNS) from ECs and transmigrating leukocytes recruited to the endothelium are major contributors to the degradation of the endothelium and EC dysfunction [22].

Mechanotransduction is the process that translates forces sensed by mechanosensors into biological signaling to modulate gene expression driving a multitude of cellular processes, including cell migration, proliferation, and differentiation. The endothelium converts mechanical forces into biological signals that trigger intracellular signaling pathways through the endothelial surface glycocalyx, activation of ion channels, receptor and nonreceptor kinases, and membrane-associated protein complexes [19].

### Glycocalyx

The glycocalyx, a negatively charged extracellular layer of proteoglycans, glycoproteins, glycosaminoglycans, heparan sulfate, hyaluronic acid, and syndecans lining the luminal surface of ECs, is highly important in endothelial function, as it plays a role in many physiological processes, such as endothelial barrier function, oxidative stress, and inflammation [22–26]. Accessibility of macromolecules to the EC surface is regulated by the overall charge distribution and structural conformation of the glycocalyx [24, 27]. Furthermore, the depth of the glycocalyx masks the interaction of leukocytes and plasma proteins with EC surface adhesion molecules, such as intercellular adhesion molecule 1 (ICAM-1), vascular cell adhesion molecule 1 (VCAM-1), as well as P- and L-Selectin. The presence of extracellular superoxide dismutase in the glycocalyx protects ECs from oxidative stress damage by quenching oxygen radicals and maintaining nitric oxide (NO) bioavailability [23]. Moreover, the glycocalyx can modulate an inflammatory response by binding or excluding certain cytokines, thereby providing or preventing access to cell surface receptors [23, 28, 29]. Thus, all these physiological functions of the glycocalyx contribute to the health of the endothelium and vascular homeostasis.

Damage to the glycocalyx occurs during IRI and contributes to endothelial dysfunction and barrier disruption [30–33]. Under normal conditions, the glycocalyx is continuously turned over, as the rate of synthesis of components of the glycocalyx equals the rate of degradation. However, IR drives the equilibrium towards degradation and shedding, thereby leading to the rapid dismantling of the glycocalyx and obstructing glycocalyx function [22, 23, 34]. In fact, degradation of the glycocalyx may be the earliest form of structural damage after IR [35, 36]. By measuring increasing levels of components of the glycocalyx, syndecan-1 and heparan sulfate, in blood at different timepoints from patients undergoing surgery of the ascending aorta with global ischemia or regional ischemia, Rehm and colleagues were the first to present evidence of IRI-mediated endothelial glycocalyx shedding in humans [37]. Furthermore, higher concentrations of syndecan-1 and heparan sulfate were also observed in coronary effluent from guinea pig hearts exposed to hypoxia-reoxygenation, thereby supporting a role for IRI in the degradation of the glycocalyx [38]. Sladden et al. showed that the presence of elevated endothelial glycocalyx breakdown products in donor lungs for transplant was associated with poor pulmonary function, reduced acceptability for lung transplant, and PGD in transplant recipients [39]. Vascular sites with deteriorated glycocalyx are more susceptible to proinflammatory effects [22].

Loss of the glycocalyx exposes cellular adhesion molecules, thus increasing leukocyte adhesion and vascular permeability with subsequent edema formation. Treatment with hydrocortisone or antithrombin III prior to IR significantly decreased the shedding of glycocalyx constituents, syndecan-1 and heparan sulfate, and inhibited the adhesion of neutrophils, thus protecting the vascular endothelium against IRI [40]. In addition, Annecke and colleagues demonstrated that protection of the endothelial glycocalyx from IRI was achieved by an inhalational anesthetic gas given before ischemia or during reperfusion [41]. This group showed that Sevoflurane preconditioning and postconditioning attenuated the release of markers of glycocalyx shedding through the inhibition of lysosomal cathepsin B secretion and preserved the structure of the glycocalyx in guinea pig hearts exposed to IR, thus proposing that Sevoflurane protected the glycocalyx from IR-mediated damage [41]. In a study comparing patients anesthetized with sevoflurane or propofol during lung resection surgery with one lung ventilation, Kim et al. discovered that the protective effects on the glycocalyx provided by sevoflurane was similar to that supplied by the intravenous anesthetic propofol [42].

Disruption of the glycocalyx contributes to neutrophil activation and adhesion, a hallmark of IRI. Neutrophils contain proteases, elastase, and proteinase-3 that degrade certain components of the glycocalyx, and the destruction of the endothelial glycocalyx has been associated with increased vascular permeability, leukocyte adhesion, and inflammation in the lungs [43, 44]. Pretreatment of guinea pig hearts subjected to 20 min of warm ischemia and 10 min of reperfusion with sevoflurane maintained the endothelium by reducing the shedding of glycocalyx constituents, which masked the adhesion molecules required for neutrophil binding [45]. Casanova and colleagues were the first to show that sevoflurane preconditioning before IRI preserved the pulmonary glycocalyx by attenuating the expression of inflammatory chemokines (MCP-1, MIP-1, and MIP-2) and leukocyte adhesion molecules (ICAM-1 and VCAM-1) in an in vivo lung autotransplant model [35]. Using a porcine lung autotransplant model, this group also demonstrated that continuous intravenous administration of lidocaine protected lungs from glycocalyx shedding, as they observed that the effects of IRI (i.e. decreased levels of syndecan-1 and heparan sulfate in lung tissue; elevated glycocalyx markers and adhesion molecules in blood; elevated levels of adhesion molecules and neutrophil activation) were significantly lower in the lidocaine cohort [36]. Moreover, mice treated with recombinant human vimentin (rhVim) prior to endotoxin exposure had significantly lower histologic acute lung injury scores compared to control mice [46]. In vitro adhesion and binding assays demonstrated that rhVim protected the vascular endothelium from neutrophil adhesion and infiltration by binding P-selectin and obstructing the interaction between P-selectin and P-selectin glycoprotein ligand-1 [46]. Although the exact mechanisms underlying glycocalyx destruction are not fully understood, these studies support a significant role for endothelial glycocalyx in IRI.

### Ion channels

Over the past decade, research has been focused on mechanosensitive ion channels, including transient receptor potential (TRP) channels and piezo channels, as potential mediators of IRI. TRP channels are non-selective transmembrane cation channels that regulate Ca^2+^ influx and signaling in the pulmonary endothelium, among other cells [47]. Activated by a number of stimuli, including sheer stress, mechanical stretch, pH, temperature, ROS, extracellular ATP, inflammatory cytokines, and membrane potential changes, the TRP family consists of seven subtypes: TRPC (canonical), TRPV (vanilloid), TRPM (melastatin), TRPML (mucolipin), TRPP (polycystin), TRPA (ankyrin), and TRPN (nitric oxide-mechanoreceptor potential C) [4, 48, 49]. Lung ECs primarily express TRPC, TRPV, TRPM, and TRPP channels.

Evidence from multiple studies demonstrates that a major contributor to lung IRI is the dysregulation of mechanotransduction-mediated TRP channels. Ca^2+^ influx through seven members of TRPC subfamily (TRPC1-7) can activate myosin light chain kinase (MLCK), inhibit cAMP generation, and reorganize cytoskeletal structures, resulting in increases in endothelial permeability, endothelial barrier failure, endothelial contraction, and subsequent extravasation of plasma macromolecules and immune cells [47, 48, 50, 51]. For example, during the early stages of lung IRI, the rise in intracellular Ca^2+^ concentration and NADPH oxidase 2 (NOX2)-derived ROS in ECs activates TRPC6, causing cell morphology changes and increasing endothelial leakage, ultimately resulting in pulmonary edema [50, 52, 53]. Stable lung weights and capillary filtration coefficient values from TRPC6 knockout mice before and after IR demonstrated that these mice were protected from IR-induced pulmonary edema [52]. Here, the specific contribution of TRPC6 to pulmonary oedema was underlined by the fact that lungs from TRPC1- and TRPC4-deficient mice were not protected from increased vascular permeability caused by IR. Furthermore, this study demonstrated that NOX2-generated ROS and phospholipase C-g-generated diacylglycerol precedes TRPC6 activation and IRI [52].

The TRPV subfamily consists of six members (TRPV1-6), of which TRPV4 is the most researched channel and has been discovered to be a crucial player in the regulation of lung endothelial barrier integrity [54, 55]. TRPV4 channels are expressed in many cell types in the lung, including ECs, alveolar epithelial cells, macrophages, and neutrophils [56], and can be activated by many forces, such as temperature, membrane stretch, oxidative and sheer stress, among others [19]. Recent studies now indicate that TRPV4 channels on ECs, immune cells, and alveolar epithelial cells mediate acute lung injury [57]. Studies have described TRPV4 channels being involved in oxidative stress-induced endothelial barrier failure in that H_2_O_2_ exposure induces Ca^2+^ influx through TRPV4 channels in murine and human lung microvascular ECs in a Fyn-dependent manner, resulting in increased endothelial permeability [58]. Using the TRPV4 agonist GSK1016790A at doses that induce injury, Villalta and colleagues observed that lung edema (total protein in bronchoalveolar lavage (BAL) fluid and lung wet-to-dry weight ratio) increased in wild-type mice [59]. Furthermore, TRPV4 activation increased protein levels of the active forms of matrix metalloproteinase 2 (MMP2) and MMP9 along with decreased expression of tissue inhibitor of metalloproteinase 2 (TIMP2), an endogenous MMP inhibitor. This was the first evidence linking the downstream effectors MMP2 and MMP9 to TRPV4-induced increases in lung permeability and edema. These data indicated that TRPV4-mediated Ca^2+^ influx elicited the activation of MMP2 and MMP9, which contributed to barrier permeability.

Our group demonstrated that endothelial cell-specific TRPV4 knockout mice as well as inhibition of TRPV4 in wild-type mice with GSK2193874 resulted in attenuation of lung dysfunction and edema following IR as demonstrated by improved oxygenation and compliance, and decreased edema, neutrophil infiltration, and proinflammatory cytokine expression [60]. This study was the first to demonstrate a role for endothelial TRPV4 in mediating edema after lung IR and highlighted the potential use of TRPV4 inhibitors as a therapeutic strategy to prevent lung IRI. Furthermore, the attenuation of proinflammatory cytokines in endothelial cell-specific TRPV4-deficient mice after IR [60] suggests that endothelial TRPV4 channels are important mediators of vascular inflammation and endothelial barrier disruption after IR.

The TRPM subfamily is a group of oxidant-activated cation channels that consists of eight members (TRPM1-8), of which TRPM2 is highly expressed in ECs. TRPM2 mediates Ca^2+^ entry in response to intracellular ADP-ribose that is generated during oxidative stress [61]. In human pulmonary artery ECs, H_2_O_2_ induces Ca^2+^ influx through TRPM2 channels, resulting in increased endothelial permeability [62]. Conditional knockout of endothelial TRPM2 in mice reduced transmigration and sequestration of neutrophils in lungs following lipopolysaccharide (LPS) challenge [63]. Although less is known about the role of TRPM2 in lung IRI, Zhong et al. demonstrated a crucial role for TRPM2-mediated ferroptosis in hepatic IRI and that pharmacological inhibition of TRPM2 may provide an effective therapeutic strategy to attenuate hepatic IR injury [64]. In addition, Khanahmad et al. showed that TRPM2 deficient mice are resistant to renal IRI and that TRPM2 channels are activated by ROS and cyclic adenosine diphosphate ribose (cADPR) generated during renal IRI [65]. These data suggest that TRPM2 channels in ECs actively mediate endothelial barrier integrity by facilitating Ca^2+^ influx by ECs and promoting the transmigration of neutrophils across the endothelium during IRI.

While TRP channels mediate Ca^2+^ influx into cells, not all Ca^2+^ influx occurs via TRP channels. Discovered in 2010, Piezo channels are non-selective cation channels that open in response to mechanical forces to allow the influx of cations, where PCa > PNa = PK [66]. Piezo channels respond to stimuli directly (e.g. mechanical stretch, chemical agonists), as well as indirectly (via other channels and upstream signals) [67]. Humans have two Piezo genes, *PIEZO1* and *PIEZO2*, and the resulting proteins share approximately 50% amino acid identity. In lungs, Piezo1 channels are highly expressed in pulmonary microvascular ECs as well as alveolar epithelial cells, and Piezo2 channels are highly expressed in airway-innervating sensory neurons [68, 69]. Three main signaling pathways have been identified downstream of Piezo1: ATP-purinergic P2X/P2Y receptors [70], Ca^2+^-dependent calpain signaling [71], and Ca^2+^-dependent TRPV4 signaling [72]. These pathways play important roles in cellular function, cell-cell communication, cell cycle progression, proliferation, apoptosis, and angiogenesis.

Endothelial Piezo1 channels have been recently shown to play a role in endothelial barrier integrity. Jiang et al. showed that high tidal volume ventilation produces excessive mechanical stretch that drastically increases the influx of Ca^2+^ through endothelial Piezo1 channels, which activates calpain signaling and promotes pulmonary endothelial hyperpermeability and edema as a result of the internalization and breakdown of VE-cadherin junctions [73]. Friedrich and colleagues used endothelial Piezo1-deficient mice to demonstrate that lung vascular hyperpermeability following increased capillary pressure is the result of Piezo1 activation and the breakdown of lung endothelial adherens junctions (AJs) and opening of paracellular routes [69]. Furthermore, the endothelial barrier was disrupted through Piezo1-dependent activation of calpain and subsequent proteolysis of VE-cadherin, b-catenin, and p120-catenin. Zhong et al. identified a novel adaptive role of EC-expressed Piezo1 in stabilizing the lung endothelial barrier in response to alveolar stretch [69]. They demonstrated that activation of Piezo1 channels in ECs elicited calcium signaling and activated calpain which cleaves Src tyrosine kinase, consequentially suppressing Src-mediated VE-cadherin phosphorylation, thereby preventing VE-cadherin internalization from AJs. Tyrosine phosphorylation of adhesion molecules by tyrosine kinases including Src may induce disassembly of catenin-cadherin complexes and microtubules [74, 75]. These findings uncovered an adaptive feedback regulation by which alveolar stretch-induced Piezo1 activation in ECs preserves the lung endothelial barrier function. Although the role of Piezo1 in IRI has not been well studied, Guo et al. hypothesized that PIEZO1 may be involved in cerebral ischemia-reperfusion injury through ferroptosis regulation [76].

### Purinergic receptors

During inflammatory injury such as IRI, cell surface pannexin 1 (Panx1) channels rapidly release ATP, a DAMP molecule, into the extracellular space, where the pro-inflammatory actions of extracellular ATP are exerted by members of the purinergic P2 receptor family such as P2X7 and P2Y2 receptors [4, 77]. A study by Sugimoto et al. in 2009 was the first to demonstrate a role of extracellular ATP in lung IRI by showing that recipient animals treated with apyrase, which hydrolyzes ATP, resulted in significant attenuation of lung IRI and vascular injury after transplantation [78]. Our laboratory recently showed that endothelial Panx1 knockout mice, and wild type mice treated with Panx1 inhibitor, are protected against vascular permeability, inflammation, and edema after lung IR, suggesting that endothelial Panx1 and efflux of ATP play important roles in the pathogenesis of lung IRI [79].

Our laboratory recently used tamoxifen-inducible, endothelial-specific purinergic P2Y2 receptor (P2Y2R), Panx1, and TRPV4 knockout mice in combination with in vivo, ex vivo, and in vitro models of IR to show that lung IR induces endothelial Panx1 channel-mediated efflux of ATP, which then activates endothelial P2Y2R signaling. In turn, this P2Y2R activation stimulated endothelial TRPV4 channel activity, resulting in endothelial barrier disruption, lung edema, leukocyte infiltration, and lung dysfunction after IR [80]. Our finding that endothelial knockout of P2Y2R reduced the expression of inflammatory cytokines in lungs after IR suggests that the endothelium may also be a source of inflammatory cytokines in lung IRI. Another possibility is that P2Y2R knockout preserves the endothelial barrier after IR, thereby dampening overall inflammation and activation of innate immune cells. This study and those described above suggest that inhibitors of components of the Panx1-P2Y2R-TRPV4 signaling axis on endothelium may be an effective therapeutic strategy to prevent or treat lung IRI via preservation of endothelial barrier function, which supports future studies toward eventual translation of these therapies to human lung transplant patients.

### Kinase signaling

Intracellular signaling pathways involving kinases in ECs have been indicated to play critical roles in IRI, where the generation of ROS and rise in intracellular Ca^2+^ through Ca^2+^ influx from plasma membrane ion channels have been shown to promote endothelial permeability and dysfunction [81–83]. The Src tyrosine kinase family member Fyn associates with the fatty acid transporter CD36 in the endothelium [81]. In addition to serving as a membrane anchor for kinase activation, data suggests that CD36 also plays a critical role in Ca^2+^ signaling, where loss of CD36 attenuates Ca^2+^ influx following thapsigargin-induced store depletion [84]. CD36 also plays an important role in ROS and Ca^2+^ signaling in the lung microvasculature. CD36 gene deficiency led to a significant attenuation of H_2_O_2_-induced Ca^2+^ influx and endothelial permeability in vitro as well as protection from lung IRI in vivo [85]. These findings suggest that loss of CD36 protects against lung IRI, likely due to CD36-mediated changes in H_2_O_2_-induced Ca^2+^ influx. Furthermore, the mechanism by which CD36 participates in this process may be by acting as a membrane scaffold for Fyn, allowing for the interactions between Fyn and TRPV4 to facilitate the phosphorylation and activation of TRPV4 in response to oxidative stress. Inhibition of another Src family member, c-Abl, protected against IRI by attenuating oxidative stress-induced endothelial barrier dysfunction and inflammation [86]. In an ex vivo IR model, lungs from New Zealand White rabbits demonstrated significantly better oxygenation and wet:dry weight ratio following IR and imatinib treatment, which inhibits c-Abl [86]. Moreover, there was remarkably less total protein in BAL fluid and infiltrating neutrophils in imatinib-treated lungs, and imatinib also abrogated DNA double-strand breaks and p53 upregulation after IR. Together, these data highlight the potential role of Src family kinases as IRI biomarkers and therapeutic targets.

Recent studies reported that p38 mitogen-activated protein kinase (MAPK) plays a role in the development of lung IRI by mediating lung endothelial barrier dysfunction. After treatment with a p38 MAPK inhibitor SB203580, left lungs of rats showed remarkably less alveolar wall thickening and inflammatory cell infiltration in an in vivo lung IRI model [87]. In addition, p38 MAPK inhibition attenuated total protein levels in BAL fluid and Evans blue dye staining, indicating that SB203580 diminished endothelial leakage after IRI. Furthermore, Western blot and immunofluorescence revealed that inhibition of p38 MAPK partially reversed the IR-induced disruption of the endothelial barrier, as expression of zonula occludens 1 (ZO-1, a key junction protein) and VE-cadherin (a critical endothelial adhesion molecule) were downregulated while ICAM-1 was upregulated [87]. Furthermore, in an in vitro IR model of lung transplantation, knockdown of p38 MAPK with small interfering RNAs in rat PMVECs reduced oxidative injury associated with inflammation, apoptosis, and cell cycle arrest [88]. Wang and colleagues demonstrated that the protective effects of p38 MAPK inhibition on IRI were enhanced when c-Jun NH_2_-terminal protein kinase was knockdown in rat PMVECs as well [89]. These studies suggest that the inhibition of p38 MAPK signaling could mitigate lung IRI-induced endothelial damage.

Sphingosine 1-Phosphate (S1P) is a sphingolipid that acts as a bioactive signal transducer, mediating intra- and extracellular signaling pathways that control endothelial barrier integrity, proliferation, cell survival, and immune cell migration [90]. ECs are the major source of S1P in the lungs [91]. Treatment of rat lung recipients with S1P prior to lung transplantation significantly improved oxygenation and lung graft wet:dry ratios compared to control recipients [92]. Furthermore, S1P treatment prevented neutrophil accumulation, reduced NF-κB-mediated proinflammatory cytokine levels (TNF-α, IL-1β, and MIP-2), and blunted the activation of Akt, p38, and JNK, indicating that S1P abrogated IRI after lung transplantation through the reduction of vascular permeability, neutrophil infiltration, proinflammatory cytokines, and EC apoptosis [92]. Our group showed that S1P-mediated protection from IRI signaled through the G-protein-coupled receptor, S1P receptor 1 (S1PR1), since a selective S1PR1 agonist, VPC01091, attenuated lung IRI and preserved endothelial barrier in mice [93]. In addition, our group found that modifying the S1P gradient (higher circulating S1P, lower tissue S1P) through the combination of S1P and sphingosine kinase 2 inhibition during ex vivo lung perfusion (EVLP) significantly improved lung compliance and vascular permeability in a murine lung IRI model [94]. While S1P and its analogues have been shown to reduce vascular leakage in small and large animal lung injury models [92–96], the clinical application is currently limited by systemic toxicity, as prolonged exposure to S1P agonists worsen vascular leakage and promote fibrosis [97]. Design of safer analogues with promising preclinical data will be required.

Rho-associated coiled-coil containing protein kinase 1 (ROCK1) is a serine/threonine kinase that is the downstream effector of the small GTPase Rho and negative regulator of endothelial barrier function [98]. In a rat lung transplant model, flushing the donor lungs with the ROCK1 inhibitor, Y-27632, prevented inflammatory cells from invading the alveolar space and decreased TNF-α levels in BAL fluid, leading to reduced pulmonary edema [99]. Furthermore, inhibition of ROCK1 by miR-144 mimetics resulted in the downregulation of ROCK1 and downstream target myosin phosphatase-targeting subunit 1 [100]. These events favor the inactivation of myosin light chain kinase, thereby stabilizing the endothelial barrier and limiting vascular leakage during lung injury. Thus, these data illustrate the important role that ROCK1 plays in the pathogenesis of lung IRI and endothelial barrier disruption.

### Integrins

Integrins, a family of cell surface receptors consisting of 24 members, each containing a single α subunit and single β subunit, regulate cellular proliferation, migration, cytokine secretion, and signaling, and thereby play critical roles in cell growth, apoptosis, inflammation, and endothelial barrier integrity [101, 102]. These receptors link the intracellular cytoskeleton with the ECM, and thus transduce external and internal mechanochemical signals across the plasma membrane upon activation after ligand binding [101]. Several studies have shown that blockade of integrin αVβ5 with inhibitory monoclonal antibodies specifically prevented increases in lung vascular permeability, which has been considered a hallmark of lung injury that is largely responsible for its characteristic pulmonary edema formation. Su et al. demonstrated that both αVβ5 genetic deletion and blocking antibody (ALULA) prevented vascular permeability after IRI in rats [103]. Furthermore, treatment of donor lungs with ALULA significantly decreased extravascular lung water, neutrophil infiltration and total protein in BAL fluid, and improved arterial oxygenation, suggesting that αVβ5 blockade prevented IRI following transplantation [104]. Zhang and colleagues examined the role of αVβ5 in IR-induced endothelial cell apoptosis and autophagy and found that αVβ5 inhibition with ALULA reduced cell permeability and cleaved caspase-3 expression after IR [105]. Moreover, ALULA treatment enhanced endothelial autophagy levels, which was reflected in the increase in LC3-II expression and decrease in p62 levels [105]. Together, these data indicated that αVβ5 inhibition mitigates IR-induced endothelial cell apoptosis, leading to attenuation of lung IRI.

## Current Therapeutics

Considerable research effort has been focused on a better understanding of the molecular mechanisms that cause lung endothelial barrier failure after IR in order to identify therapeutic targets to treat or prevent IRI. One of the endothelial protection methods that is often implemented in many transplantation clinics to reduce lung IRI and improve lung function is the “controlled reperfusion” or regulated reestablishment of blood flow during the early stages of reperfusion, combined with controlled ventilation to prevent hypercapnia [106, 107]. Several novel strategies have emerged over the past years which show considerable promise for preservation or reconstitution of endothelial barrier function in vitro and in preclinical trials. However, there are no current therapeutic agents yet clinically available to prevent or treat IRI, and treatment strategies are limited to maintaining lung function (Table 1).

**Table 1:** Potential therapies for lung ischemia-reperfusion injury

Potential Therapies	Targets	References
Controlled reperfusion and ventilation	Endothelium (general)	106, 107
Sevoflurane	Glycocalyx	35, 108
Interferon-β-1a	Multiple cells including ECs	109
GSK2193874	TRPV4	110, 111
GSK2220961	TRPV4	54
GSK2337429	TRPV4	54
GSK2798745	TRPV4	112–114
HC-067047	TRPV4	111
Mibefradil	T-type Ca^2+^ channels	115
Flunarizine	T-type Ca^2+^ channels	115
Probenecid	Panx1	79
Carbenoxolone	Panx1	79
Imatinib	Abl tyrosine kinase	116, 117
3-methyladenine	Autophagy	118–120
S1P	S1P receptor 1	92–97
EVLP	Endothelium (general)	121–123
EVLP + A2AR agonists	Endothelium (general), A2AR	121–127
EVLP + A2BR agonists	Endothelium (general), A2BR	128–129
EVLP + S1P and SK inhibitors	Endothelium (general), S1PR, SK	94

*Abbreviations*: A2AR, adenosine 2 A receptor; A2BR, adenosine 2B receptor; EVLP, ex vivo lung perfusion; Panx1, pannexin 1; S1P, sphingosine-1-phosphate; SK, sphingosine kinase; TRPV4, transient receptor potential cation channel subfamily V member 4

Since IRI involves glycocalyx damage, several therapeutics have been investigated, either targeted to the prevention of glycocalyx injury or restoration of the ultrastructure of the endothelium. Sevoflurane is an anesthetic and potent vasodilator that protects the endothelium following IR. Blood from sevoflurane-anesthetized pigs subjected to IRI (induced by a balloon catheter in the thoracic aorta) had low levels of negatively charged heparan sulfate compared to blood from propofol-anesthetized pigs, signifying a healthy glycocalyx [108]. In an in vivo lung autotransplant model in pigs, sevoflurane pretreatment preserved the pulmonary glycocalyx, reduced serum levels of heparan sulfate and syndecan, and decreased expression of adhesion molecules and chemokines [35].

A phase I clinical trial demonstrated mortality benefit in acute respiratory distress syndrome treated with interferon-β-1a [109]. The proposed mechanisms of benefit were modulation of inflammation (possibly neutrophil endothelial interactions) and endothelial barrier function via CD73-mediated dephosphorylation of AMP. While non-randomized, the mortality benefit (24% absolute reduction) in this study suggests that targeting the lung endothelium may hold promise as a viable therapeutic strategy in IRI.

An increase in intracellular calcium not only increases the permeability of ECs, but also contributes to neutrophil activation and EC inflammation. Since calcium influx plays a crucial role in the occurrence and development of lung IRI, blocking calcium influx into ECs may be an effective therapeutic strategy for lung injury. An orally active TRPV4 antagonist, GSK2193874, was effective in inhibiting lung edema induced by heart failure [110]. Two other TRPV4 inhibitors (GSK2220961, GSK2337429) ameliorated acute lung injury in mice when administered 30 min after exposure to hydrochloric acid or chlorine gas [54]. When GSK2193874 was administered 20 min before the induction of acute lung injury by acid instillation, key hallmarks of acute lung injury (i.e. lung edema, inflammation, poor gas exchange, lung dysfunction, and neutrophil infiltration) were attenuated in mice [111]. However, another TRPV4 inhibitor, HC-067047, applied 45 min after lung injury to simulate a clinically more relevant scenario, offered no protection from acute lung injury, suggesting that timing of treatment may be important in the effectiveness of TRPV4 inhibition in preventing IRI [111]. Blocking TRPV4 (e.g. with GSK2798745) was also proposed as a promising and feasible approach to protecting the alveolo-capillary barrier in lungs of Covid-19 patients [112]. In the first human clinical trial (NCT03511105), LPS-induced elevation of total protein and neutrophils in BAL fluid from the airway site after application of GSK2798745 was not different in comparison to placebo-treated controls [113, 114]. However, the effectiveness of TRPV4 inhibition as a strategy to treat lung IRI after transplantation has not yet been tested. Therefore, although modulating TRPV4 channel activity may be useful as a therapeutic approach for lung IRI, cell type and injury model should be considered in order to establish clinically successful drugs.

Mibefradil and Flunarizine, two T-type Ca^2+^ channel blockers, significantly decreased LPS-induced total cell number, protein concentration, and Evans blue dye extravasation in the lung, as well as TNF-α and IL-6 levels in BAL fluid [115]. In addition, Mibefradil also attenuated pathological alterations in lung tissue of LPS-challenged mice and suppressed the activity of MPO and NF-κB, a central transcription factor regulating gene expression of various inflammatory mediators [115]. Since influx of inflammatory cells, protein leakage, and cytokine storms are crucial events of IRI, these Ca^2+^ channel inhibitors may provide protection against IR injury.

Tyrosine kinase inhibitor imatinib, which was originally developed to inhibit the chronic myelogenous leukemia-causing BCR-Abl fusion protein, has been shown to attenuate pulmonary vascular permeability induced by a broad range of mediators in a clinically relevant murine model of ARDS [116]. Imatinib also attenuated thrombin and histamine-induced barrier dysfunction in vitro [117]. Given its multiple sites of action, further mechanistic work is required to progress imatinib as a potential therapy in ARDS. We propose that imatinib may be a therapeutic option to prevent or treat lung IRI, which warrants further investigation.

Autophagy was implicated to be involved in the pathogenesis of lung IRI, as the inhibition of autophagy by 3-methyladenine (3-MA) resulted in reduced edema, oxidative stress, and neutrophil activation in a rat lung IRI model [118]. Furthermore, Liu et al. demonstrated that 3-MA inhibited apoptosis and enhanced proliferation to protect against IR-induced lung injury in a rat model of orthotopic left lung transplantation [119]. However, another study showed that promoting autophagy with rapamycin in human pulmonary microvascular endothelial cells and mice ameliorated tight junction damage and cell death, suggesting that autophagy was protective against IRI [120]. While there seems to be a paucity of research on the role autophagy plays in lung IRI, these studies advocate for more research on whether autophagy preserves or damages the endothelium.

The important, recent development of ex vivo lung perfusion (EVLP) has allowed the use of marginal donor lungs, such as donation after cardiac death (DCD) lungs, to help resolve the shortage of available donor lungs for transplantation. Steen and colleagues developed the “Lund protocol” that utilized a special perfusion solution (Steen solution) aimed directly at preserving the endothelium to reduce edema, and were the first group to successfully transplant DCD lungs [121]. Furthermore, they were also able to perform transplantations with marginal donor lungs using EVLP and Steen solution [122]. A group from Toronto modified the perfusion flow, perfusate composition and temperature, and respiratory rate, in the Lund protocol to create the “Toronto protocol” and successfully transplanted 372 EVLP-rehabilitated marginal donor lungs and DCD lungs [123]. These studies have encouraged the idea that EVLP can be used as a platform for therapeutic treatment to further recondition lungs to prevent primary graft dysfunction. Our group showed that EVLP with Steen solution supplemented with an adenosine A_2A_ receptor (A2AR) agonist significantly reduced pulmonary edema and interferon-γ and dramatically improved the oxygenation index, suggesting that acute IRI was attenuated in porcine lungs undergoing EVLP with A2AR-supplemented Steen solution [124]. This combination of EVLP and A2AR agonists has also been demonstrated to recondition DCD lungs for lung transplantation in preclinical murine and porcine models [125–127]. Furthermore, we determined that adenosine A_2B_ receptor antagonist treatment during EVLP significantly attenuated lung dysfunction, interleukin-8 production, and vascular permeability in a murine lung IRI model [128] as well as enabled successful transplantation of porcine DCD lungs [129]. As mentioned above, the combination of S1P and sphingosine kinase inhibitors during EVLP provided endothelial protection to improve lung function in a murine DCD model [94]. These studies give support for the use of EVLP in combination with therapies targeted towards the many mediators of IRI (especially to block endothelial barrier dysfunction) to prevent the detrimental effects of lung IRI.

## Conclusion

Lung IRI is a complex, rapid and robust inflammatory response marked by lung dysfunction, endothelial barrier disruption, oxidative stress, increased vascular permeability, edema, alveolar damage, and neutrophil infiltration. Two key events during IRI are vascular permeability and neutrophil infiltration, which results in severe edema and neutrophil-mediated injury. Each of these events are connected and dependent on the health of the endothelium, consequently leading to endothelial barrier dysfunction and edema, a principal hallmark of IRI. Although early studies on mechanisms of lung IRI focused on the innate immune responses, more recent studies have focused more on the endothelium, which is beginning to unravel the multi-factorial pathogenesis and immunologic mechanisms underlying IRI. Many important mediators of endothelial dysfunction after IR have now been identified, as reviewed here (Fig. 1). Spanning from the extracellular glycocalyx to ion channels and purinergic receptors to kinases and possibly many others yet to be discovered, these mediators of endothelial barrier integrity and IRI may serve as prospective targets for lung IRI therapy. While there are no definitive clinical therapies currently available, the accumulating evidence on the critical role the endothelial barrier plays in IRI suggests that promising treatments targeting the endothelium may be approaching soon. The ability to halt this rapid and potentially fatal injury could be significantly enhanced through the application of endothelial-directed therapies, as well as combination therapies, where more than one signaling pathway may be targeted.

## Data Availability

No datasets were generated or analysed during the current study.

## Abbreviations

ARDS: Acute respiratory distress syndrome
ATP: Adenosine triphosphate
AJs: Adherens junctions
BAL: Bronchoalveolar lavage
BCR-Abl: Breakpoint cluster region-Abelson
DAMPs: Damage-associated molecular patterns
EC: Endothelial cell
EVLP: Ex vivo lung perfusion
ECM: Extracellular matrix
ICAM1: Intercellular adhesion molecule 1
IL: Interleukin
IRI: Ischemia-reperfusion injury
KGF-2: Keratinocyte growth factor-2
MCP-1: Monocyte chemotactic protein 1
MIP-1: Macrophage inflammatory protein 1
MIP-2: Macrophage inflammatory protein 2
MMP2: Matrix metalloproteinase 2
MAPK: Mitogen-activated protein kinase
NOX2: NADPH oxidase 2
NO: Nitric oxide
PGD: Primary graft dysfunction
rhVim: Recombinant human vimentin
RNS: reactive nitrogen species
ROS: Reactive oxygen species
ROCK1: Rho associated coiled-coil containing protein kinase 1
SARS-CoV2: Severe acute respiratory syndrome coronavirus type 2
S1P: Sphingosine 1-phosphate
TRP: Transient receptor potential
TRPA: ankyrin
TRPC: Transient receptor potential canonical
TRPM: Transient receptor potential melastatin
TRPML: Transient receptor potential mucolipin
TRPN: Transient receptor potential nitric oxide-mechanoreceptor potential C
TRPP: Transient receptor potential polycystin
TRPV: Transient receptor potential vanilloid
VCAM1: Vascular cell adhesion molecule 1
